# βArrestins in Cardiac G Protein-Coupled Receptor Signaling and Function: Partners in Crime or “Good Cop, Bad Cop”?

**DOI:** 10.3390/ijms141224726

**Published:** 2013-12-18

**Authors:** Anastasios Lymperopoulos, Shmuel Negussie

**Affiliations:** The Laboratory for the Study of Neurohormonal Control of the Circulation, Department of Pharmaceutical Sciences, Nova Southeastern University College of Pharmacy, Fort Lauderdale, FL 33328, USA

**Keywords:** βarrestins, signal transduction, GPCR, heart, adrenergic receptors, angiotensin II receptors, desensitization

## Abstract

βarrestin (βarr)-1 and -2 (βarrs) (or Arrestin-2 and -3, respectively) are universal G protein-coupled receptor (GPCR) adapter proteins expressed abundantly in extra-retinal tissues, including the myocardium. Both were discovered in the lab of the 2012 Nobel Prize in Chemistry co-laureate Robert Lefkowitz, initially as terminators of signaling from the β-adrenergic receptor (βAR), a process known as functional desensitization. They are now known to switch GPCR signaling from G protein-dependent to G protein-independent, which, in the case of βARs and angiotensin II type 1 receptor (AT_1_R), might be beneficial, e.g., anti-apoptotic, for the heart. However, the specific role(s) of each βarr isoform in cardiac GPCR signaling and function (or dysfunction in disease), remain unknown. The current consensus is that, whereas both βarr isoforms can desensitize and internalize cardiac GPCRs, they play quite different (even opposing in certain instances) roles in the G protein-independent signaling pathways they initiate in the cardiovascular system, including in the myocardium. The present review will discuss the current knowledge in the field of βarrs and their roles in GPCR signaling and function in the heart, focusing on the three most important, for cardiac physiology, GPCR types (β_1_AR, β_2_AR & AT_1_R), and will also highlight important questions that currently remain unanswered.

## 1. General Considerations of βarrs

The G protein-coupled receptors (GPCRs) or heptahelical or seven-transmembrane spanning receptors are by far the largest and most diverse superfamily of cell surface receptors. Approximately 600 distinct genes encoding non-olfactory GPCRs make up greater than 1% of the human genome [1]. Such evolutionary diversity enables GPCRs to detect an extraordinary array of extracellular stimuli. GPCRs function in neurotransmission, neuroendocrine control of physiologic homeostasis and reproduction, and regulation of hemodynamics and intermediary metabolism, and they control the growth, proliferation, differentiation, and death of multiple cell types. It is estimated that more than half of all drugs in clinical use target GPCRs, acting either to mimic endogenous GPCR ligands, to block ligand access to the receptor, or to modulate ligand production [1]. The importance of this receptor superfamily was highlighted last year by the awarding of the two pioneers of the field, Bob Lefkowitz and Brian Kobilka, with the Nobel Prize in Chemistry [2]. Among the various organ systems, whose physiology and homeostasis GPCRs regulate, perhaps the most prominent is the cardiovascular system, including the heart *per se*. For instance, cardiac function (contractility) is tightly controlled by the activity of β-adrenergic receptors (β_1_- and β_2_ARs) located in the membranes of cardiac myocytes [3]. Cardiac structure and morphology are regulated by angiotensin II (AngII) type 1 receptors (AT_1_Rs) present (mainly) in cardiac fibroblast and endothelial cell membranes, but also, to a lesser extent, in cardiomyocyte membranes [4]. Moreover, the neurohormonal control of the circulation, e.g., catecholamine and corticosteroid release by the adrenal glands or activation of the renin-angiotensin-aldosterone system (RAAS) by the juxtagomerular apparatus of the kidneys, is also under tight regulation by various GPCRs [5].

Agonist binding of all these receptors promotes their interaction with heterotrimeric G proteins, which initiates the classical intracellular signaling of these receptors that ultimately leads to a variety of cellular responses/physiological effects. At the same time though, agonist binding promotes the phenomenon of homologous or agonist-dependent receptor desensitization, which is the molecular basis of the waning of the cellular responsiveness to persistent receptor stimulation and constitutes a major classical homeostatic mechanism of cellular physiology [6]. This agonist-dependent desensitization is conferred, at the molecular level, by phosphorylation of the receptor by the family of kinases known as G protein-coupled receptor kinases (GRKs). GRK2 is the most prominent member of this family, binding to and phosphorylating a vast majority of GPCRs, including cardiac βARs and AT_1_Rs [7]. It is also the most abundant GRK isoform in the heart and in the cardiovascular system in general [7]. GRK-dependent phosphorylation enhances the affinity of the receptor for binding to the ubiquitous receptor adapter proteins βarrestins (βarrs), which comprise two isoforms in mammals, βarr1 and -2, also known as arrestin (Arr)-2 and -3, respectively [6]. The βarrs are also abundantly expressed in the heart and in the vasculature, as well as in several other tissues [1,6]. βarr1 and -2 bind directly to GRK-phosphorylated GPCRs, forming a stoichiometric complex that is stereochemically incapable of further G protein coupling (a phenomenon referred to as “functional desensitization” of a GPCR). βarr activation occurs when the polar core located in the hinge region between the two globular domains of the βarr molecule interacts with GRK-phosphorylated residues on the receptor, displacing the βarr *C*-terminus and exposing the concave surface of the globular domains to interact with the receptor [8]. Receptor binding produces significant conformational changes in the βarr molecule [9], whereas, conversely, βarr binding stabilizes a high-agonist affinity state of the receptor, prompting some authors to characterize the receptor-βarr complex as an “alternative ternary complex” analogous to the ternary complex existing between agonist-receptor-G protein in the absence of GTP [10]. βarrs (contrary to their retinal system-residing counterparts, the visual Arrs) further dampen G protein signaling by linking receptors to the clathrin-dependent endocytic machinery [1,11]. The *C*-terminus of βarrs directly binds clathrin heavy chain and the β2 adaptin subunit of the AP-2 complex, two intergral components of the endocytic machinery, as it gets displaced by the engagement of the receptor [12]. Clathrin/AP-2 binding causes βarr-bound GPCRs to cluster in clathrin-coated pits, which are pinched off the plasma membrane by the motor protein dynamin. This βarr-dependent endocytosis (receptor internalization or sequestration) removes receptors from the cell surface, rendering them less responsive to subsequent stimuli. From that point on, most GPCRs fall into one of two classes based on their affinity for the two βarr isoforms and the longevity of the receptor-βarr interaction [13]. One class exhibits higher affinity for βarr2 than βarr1 and forms transient receptor-βarr complexes that dissociate soon after the receptor internalizes. These receptors (e.g., the β_2_AR) rapidly recycle back to the plasma membrane ready to signal again upon the next encounter with agonist (receptor resensitization). The other class exhibits equivalent affinities for βarr1 or -2 and forms more stable receptor-βarr complexes that remain intact as the receptor undergoes endosomal sorting. These receptors (e.g., AT_1_R & vasopressin V_2_ receptor) are sequestered in endosomes and tend to recycle slowly or undergo lysosomal targeting for degradation (receptor downregulation, *i.e.*, total cellular receptor number reduced).

Unlike the catalytic interaction of a GPCR with its cognate G protein, GPCRs form relatively stable complexes with βarrs that persist on a time scale of minutes to hours [14]. It was the discovery that βarrs serve as adapters not only in the context of GPCR endocytosis but also in linking activated receptors to other enzymatic effectors that ushered in a new paradigm shift in GPCR signal transduction [6,11]. It is nowadays well known that βarrs bind a number of catalytically active proteins and recruit them to agonist-occupied GPCRs, among them Src family tyrosine kinases, components of the ERK1/2 and c-Jun *N*-terminal kinase 3 (JNK3) mitogen-activated protein (MAP) kinase cascades, the E3 ubiquitin ligase Mdm2, the cAMP phosphodiesterases (PDE) PDE4D3/5, diacylglycerol kinase (DGK), the inhibitor of nuclear factor (NF)-κB IκBα, and the Ser/Thr protein phosphatase (PP) PP2A [6,11]. It is via these interactions that βarr binding to the receptor initiates secondary waves of signal transduction independently of G proteins, usually as the GPCR-βarr complex travels through endosomal compartments during its endocytosis [6]. Thus, in addition to GPCR desensitization (G protein uncoupling) and internalization, βarrs perform a third very important biological function in cells: Signal transduction from GPCRs, *i.e.*, in essence switching of the signaling of the receptor from G protein-dependent to G protein-independent [11]. Of course, βarr-mediated signaling is qualitatively different from the G protein-mediated signal transduction; for instance, βarr signaling does not result in signal amplification, as it usually proceeds through a 1:1 stoichiometry [6]. Additionally, it exhibits specific subcellular localization, dictated by the location of the βarr-based molecular signaling scaffold, which significantly affects the ultimate signaling events produced, *i.e.*, βarrs provide compartmentalization to the signaling cascade they induce, whereas G protein signaling proceeds (“diffuses”) throughout the cell all the way down to the cell nucleus [6,11].

Given the importance of GPCRs in regulation of cardiovascular homeostasis touched upon above, it comes as no surprise that the two βarrs play very important roles in regulation of cardiovascular (and, in particular, of cardiac) physiology and homeostasis, as well [1]. The cardiovascular roles of βarrs have only recently started to become elucidated thanks to a combination of techniques and tools, such as the utilization of the (global) βarr1 and βarr2 knockout (KO) mice, usage of isoform-specific siRNA knockdown in *in vitro* systems, employment of *in vitro* and *in vivo* systems of artificially constructed GPCR mutants incapable of signaling through G proteins, and, finally, synthesis and development of signaling pathway-selective (biased) GPCR ligands that preferentially elicit βarr signaling over G protein signaling (or *vice versa*). The present review will focus on the roles the two mammalian βarrs play in GPCR signaling and function specifically in the heart. We will start with an overview of what is currently known about the physiology/pathophysiology of the two cardiac βarrs, and then discuss topics pertaining to cardiac βarrs that are currently under intense investigation, highlighting particular areas and questions that await elucidation and answers.

## 2. Cardiac β_1_AR Signaling and βarrs

The principal role of βARs in the heart is regulation of cardiac rate and contractility in response to the catecholamines [3]. Out of the three known mammalian βAR subtypes, the β_1_AR is the predominant one in cardiac myocytes, representing 75%–80% of total βAR density, followed by the β_2_AR, which comprises about 15%–18% of total cardiomyocyte βARs and the remaining 2%–3% is β_3_ARs (under normal conditions) [5]. β_1_AR stimulation by catecholamines results in the dissociation of the stimulatory G protein alpha subunit (Gα_s_) from G_βγ_. Gα_s_ stimulates adenylyl cyclase (AC) to produce 3′-5′-adenosine monophosphate (cyclic AMP, or cAMP), which in turn, by activating protein kinase A (PKA), regulates different intracellular, sarcolemmal and myofibrillar substrates, thus exerting the cellular effects of β_1_AR activation on cardiac chronotropy, inotropy and lusitropy. In addition, G_βγ_ can also activate downstream effectors that participate in cardiac signaling regulation [3]. β_2_AR also mediates the effects of catecholamines on the heart, but in a qualitatively different manner from β_1_AR, as it can also couple to the AC inhibitory G protein (G_i_). It is now generally accepted that in the heart, β_2_AR signals and functions in a substantially different way compared to β_1_AR [15–17]. Importantly, whereas β_1_AR activation enhances cardiomyocyte apoptosis, β_2_AR exerts beneficial anti-apoptotic effects in the heart, purportedly through this G_i_-mediated signaling [15–17]. Several studies using transgenic mice, β_2_AR-selective stimulation and adenoviral-mediated β_2_AR overexpression have established a consensus that β_2_AR signaling is predominantly cardio-protective, improving cardiac function and decreasing apoptosis, whereas β_1_AR-elicited signaling has detrimental effects in the heart [18,19]. Of note, the differences between these two predominant cardiac βARs in terms of signaling properties might take a quite different shape and have a much bigger bearing on pathophysiologic implications in the setting of heart failure (HF): for instance, β_1_AR is selectively down-regulated (*i.e*., total cellular receptor number reduced) in HF, thus shifting the above mentioned stoichiometry of β_1_AR:β_2_AR towards 50:50 in the failing heart from 75:~25 in the normal, healthy heart [3]. However, β_2_AR is also non-functional and does not signal properly in the failing heart [3]. In addition, β_2_AR signaling in the failing heart might differ substantially from the beneficial signaling pattern of the normal heart; it seems to be more “diffuse” (less “compartmentalized”) and resembles more the pro-apoptotic cAMP signaling pattern of its β_1_AR counterpart in HF [5]. Therefore, the aforementioned stoichiometric shift in favor of the “good” β_2_AR in HF appears unable to help the heart improve its structure and function.

As co-factors of GRKs in cardiomyocyte β_1_AR desensitization and downregulation, βarrs contribute to the diminished inotropic and adrenergic reserves of the failing heart and their inhibition should theoretically be beneficial in acute HF, as it would enhance the Gα_s_-AC-PKA-mediated pro-contractile signaling of cardiac βARs, which increases cardiac contractility [3]. Studies in (global) βarr1KO mice have confirmed that cardiac βarr1 diminishes inotropic and adrenergic reserves by means of desensitizing cardiomyocyte β_1_- and β_2_ARs, since contractility of βarr1KO mice in response to isoproterenol, a βAR agonist cardio-stimulant (positive inotrope), is significantly augmented compared to wild type mice (but basal contractility is unaffected) [20]. Of note, cardiac βarr2 does not appear to be up-regulated compensatively in these βarr1KO mice and to compensate for the loss of βarr1 in the myocardium [20], strongly indicating that the two βarr isoforms are rarely (if at all) physiologically interchangeable. In HF, chronic catecholaminergic stimulation of the β_1_AR promotes cardiac hypertrophy, decreases contractility, and increases myocyte apoptosis [21]. As a result, administration of β-blockers is currently part of standard care in the clinical management of congestive HF [22,23]. βarrs have been shown *in vitro* to mediate the mitogenic signaling of EGF (Epidermal Growth Factor) receptor (EGFR) transactivation by the β_1_AR [24]. As inhibition of EGFR contributes to dilated cardiomyopathy, β_1_AR signaling via βarr2 appears to be protective rather than deleterious for the heart and βarr2-dependent EGFR transactivation might exert a cardio-protective effect [25,26]. The physiologic relevance of EGFR transactivation by β_1_AR-bound βarr2 has been demonstrated in transgenic mice overexpressing wild-type β_1_ARs or mutant β_1_ARs lacking GRK phosphorylation sites and thus unable to undergo GRK-mediated phosphorylation and bind βarrs [24]. Under conditions of chronic catecholamine stress, transgenic mice overexpressing the mutant β_1_AR are incapable of transactivating cardiac EGFRs and show marked myocyte apoptosis and left ventricular dilatation compared to mice expressing wild-type β_1_ARs [24]. Further substantiating a role for EGFR transactivation in the mechanism underlying these findings, pretreatment of mice overexpressing the wild-type β_1_AR in their hearts with the known selective EGFR inhibitor (and anti-cancer drug) erlotinib prevents any improvement in cardiac function upon chronic stimulation with catecholamines [24].

**Figure 1.**
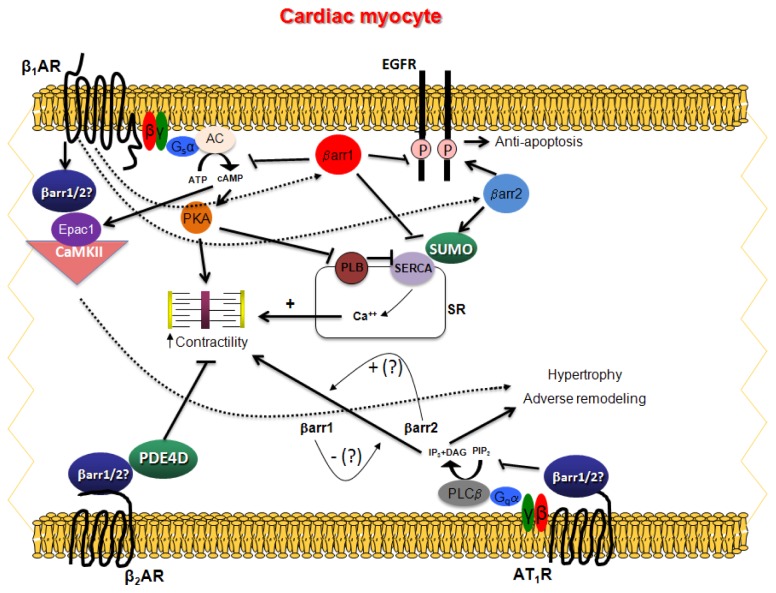
Schematic illustration of some important signaling pathways inside a cardiac myocyte affected by the two βarrs. SR, sarcoplasmic reticulum; PLB, phospholamban; ATP, adenosine triphosphate; PLCβ, phospholipase C-beta; IP_3_, inositol (1,4,5)-trisphosphate; DAG, 2-diacylglycerol; PIP_2_, phosphatidylinositol (4,5)-bisphosphate. See text for all other acronyms and details. “?” indicates that the suggested effect is currently under investigation, “βarr1/2?” indicates that which one of the two βarrs mediates the suggested effect (or if both βarrs do) is currently unknown.

Very recently, our laboratory was able to delineate the specific roles of cardiac βarr1 in βAR signaling and function during post-myocardial infarction (MI) HF progression by studying the βarr1KO mice after MI [27]. More specifically, we found that cardiac βarr1 (and not βarr2) is the βarr isoform responsible for the aforementioned βAR desensitization and downregulation (Figure 1), the molecular hallmark of HF, since βarr1KO mice have significantly better cardiac β-adrenergic and inotropic reserves, accompanied by elevated cardiac functional βAR numbers and cAMP pro-contractile signaling, compared to control, age-matched wild type mice, both in healthy conditions and, even more so, after MI. Notably, cardiac βarr2 did not compensate for these phenotypic effects of cardiac βarr1 absence [27]. In addition, the genetic deletion of βarr1 resulted in reduced cardiac apoptosis, inflammation, and adverse remodeling post-MI [27]. Importantly, and with regards to cardiac βarr-dependent signaling, we uncovered that βarr2 is the βarr isoform that mediates the beneficial, anti-apoptotic EGFR transactivation by the β_1_AR in the heart mentioned above, and that cardiac βarr1 actually antagonizes βarr2 for this effect (Figure 1), since βarr1KO mouse hearts display significantly elevated EGFR activity levels in response to β_1_AR-selective stimulation *in vivo* (treatment with isoproterenol in the presence of the β_2_AR-selective antagonist ICI 118,551), compared to control, wild type mouse hearts, both under healthy conditions and post-MI [27]. Another interesting finding from our study of βarr1KO mice was that cardiac sarco (endoplasmic) reticulum Ca^2+^-ATPase (SERCA)-2a activity of these mice was elevated, compared to control wild type mice, again both under healthy conditions and post-MI [27]. SERCA2a is an essential positive regulator of cardiac contractility activated by βARs (normally via PKA) [28]. Elevated activity of this cardiomyocyte calcium pump in post-MI βarr1KO mice was expected, since cardiac βarr1 desensitizes and downregulates βARs in post-MI HF. The fact that it was found elevated also in normal, healthy βarr1KO mice however, whose cardiac β-adrenergic reserve is no different from that of control, wild type mice, raises the interesting possibility that cardiac βarr1 suppresses SERCA2a activity not only indirectly (by desensitizing βARs) but also directly [27]. Indeed, currently ongoing studies in our laboratory indicate that cardiac βarr2 is capable of interacting with SERCA2a, increasing its SUMO (small ubiquitin-like modifier)-ylation and activity [29], and cardiac βarr1 might normally antagonize this [30] (Figure 1). Taken together, all of the above studies seem to establish a consensus for cardiac β_1_AR signaling, according to which: (a) β_1_AR signaling is beneficial for the heart when it is mediated by βarrs (and specifically βarr2), and cardiotoxic (pro-apoptotic) when it is mediated by G proteins; and (b) β_1_AR-boundβarr1 is detrimental for cardiac function (diminishes contractility, enhances apoptosis and inflammation, antagonizes βarr2-dependent beneficial signaling), whereas β_1_AR-boundβarr2 is (mostly) beneficial for the heart, courtesy of its positive signaling effects (EGFR transactivation, SERCA2a potentiation) (Figure 1).

However, this consensus was recently challenged by a study by Rockman and colleagues, in which cardiac βarrs were shown to be involved in cardiac β_1_AR-induced activation of Ca^2+^/Calmodulin Kinase II (CaMKII) [31]. CaMKII is known to induce myocyte hypertrophy and apoptosis in the heart, thereby playing a detrimental role in HF pathophysiology [32]. Both βarrs were shown to orchestrate a multi-protein scaffold comprising β_1_AR, CaMKII and Epac (Exchange protein directly activated by cAMP)-1 in mouse hearts *in vivo*, which leads to enhanced downstream cardiac CaMKII signaling [31] (Figure 1). This multi-molecular complex appears to form under basal conditions and β_1_AR activation (e.g., with isoproterenol) enhances its formation [31]. Importantly, β_2_AR does not seem capable of forming this complex, its formation is not affected by PKA and, finally, in contrast to EGFR transactivation (see above), both βarr1 and βarr2 appear capable of scaffolding this complex [31] (Figure 1). Certainly, these findings warrant further confirmation, but, if proven to hold true across species, they could potentially have enormous implications for cardiac β_1_AR pathophysiology; since CaMKII signaling is generally damaging for the heart (pro-apoptotic, pro-arrhythmic, *etc.*), this appears to be a cardiotoxic effect of cardiac β_1_AR βarr-dependent signaling, contrary to the EGFR transactivation by the same GPCR and via cardiac βarrs which is postulated to be beneficial in the heart (see above). On the other hand, if cardiac βarrs can mediate β_1_AR-inducedEpac1 activation (Figure 1), then this would mean that cardiac βarrs cannot terminate the cAMP-mediated signaling of the cardiac β_1_AR completely, despite the fact they uncouple the receptor from the G proteins; they merely terminate specifically its PKA-dependent signaling only.

## 3. Cardiac β_2_AR Signaling and βarrs

Since βarrs can desensitize (uncouple from G proteins) the cardiac β_2_AR, as well, βarr binding to this receptor subtype in the heart should also be (predominantly) deleterious, as both the G_s_ protein-mediated, pro-contractile signaling and the G_i/o_ protein-mediated, anti-apoptotic signaling of the cardiac β_2_AR (see above) are inhibited [33]. However, βarr-dependent signaling stimulated by the β_2_AR can exert some beneficial effects in the cardiac myocyte, as it can be anti-apoptotic and also anti-inflammatory in its own right, e.g., by promoting ERK activation which increases cardiomyocyte survival and proliferation, and by blocking NF-κB activation which leads to pro-inflammatory cytokine production [34–36]. In contrast to the β_1_AR, which forms a complex with PDE4D8 directly (without βarr involvement) when inactive and gets disassembled upon agonist binding, the β_2_AR can form, thanks to the βarrs, a complex with another PDE variant, PDE4D5, upon its agonist activation [37] (Figure 1). ThisPDE4D5 recruitment to the β_2_AR plays a crucial role in compartmentalizing the generated cAMP signal, posing a “brake” on the ability of cAMP to stimulate contractility [38] (Figure 1). Working in mouse hearts that lack β_1_ARs but express the known GRK2 inhibitor mini-gene βARKct (β_1_AR^−/−^/βARKct), we found that this βarr-dependent PDE4D recruitment to the cardiac β_2_AR is dependent on GRK2 and that it is, indeed, sufficient to prevent this βAR subtype from signaling to increased contractility in the heart *in vivo* [39]. GRK2 inhibition with βARKct in the hearts of β_1_AR KO mice proved essential and capable of allowing these hearts to increase their contractile function in response to catecholaminergic stimulation, all the while diminishing the interaction of PDE4D with the cardiac β_2_AR *in vivo* at the same time [39]. Thus, cardiac βarrs seem to impede β_2_AR pro-contractile signaling *in vivo*, not only by uncoupling the receptor from the G_s_ protein-cAMP signaling pathway, but also by recruiting PDE4D to the cardiac β_2_AR (following its agonist-dependent phosphorylation by GRK2). Which one of the two βarrs normally mediates this effect or whether both do remains an open question worth investigating in the future (Figure 1). In heterologous, transfected cell systems however, both βarrs are capable of binding PDE4D and of recruiting it to the β_2_AR [40].

Of note, several β_2_AR ligands (the majority of them β-blockers, very useful in cardiovascular practice) have been tested at their ability to stimulate βarr signaling *vs.* G protein signaling from the β_2_AR (*i.e*., “bias” towards βarrs) [41]. Using ERK activation as a readout in transfected HEK293 cells, isoetharine and carvedilol have been identified as βarr “biased” agonists at the β_2_AR (*i.e*., activate βarr signaling from the receptor without eliciting G protein activation) [42]. This finding has been postulated to be part of the mechanism of carvedilol’s beneficial effects in HF [23]; however, it awaits confirmation with *in vivo* studies specifically on cardiac β_2_AR, and, once confirmed, it would be interesting to delineate whether carvedilol preferentially stimulates the binding to the cardiac β_2_ARs (and β_1_ARs) of one βarr isoform over the other or the binding of both cardiac βarrs is equally induced.

## 4. Cardiac AT_1_R Signaling and βarrs

In the heart, the AT_1_R is (mainly) expressed in cardiac fibroblasts, where it stimulates cellular proliferation thus promoting fibrosis, and in cardiac myocytes, where it again stimulates growth thus promoting cardiac hypertrophy [43]. Whether it can also promote cardiomyocyte contractility however, is still a matter of debate [43]. Nonetheless, combined with other cellular effects leading to inflammation and oxidative stress development in the heart, cardiac AT_1_R effects are clearly maladaptive and damaging for both the structure and function of the cardiac muscle, playing a pivotal role in the so-called adverse remodeling of the post-MI heart progressing to HF [4,43]. AT_1_R is a classic G_q/11_-coupled receptor that can also couple to G_i/o_ proteins [4]. With regards to their classical role as G protein-dependent signaling terminators (desensitizers), very little is known about βarrs and AT_1_Rs and even less about cardiac βarrs and AT_1_Rs. The AT_1_R is a known GRK substrate, thus cardiac βarrs are bound to confer its desensitization in the heart secondary to its phosphorylation by GRKs, which has been demonstrated *in vivo* [4]. However, βarr-mediated AT_1_R desensitization *per se* has never been directly investigated *in vivo*. Intriguingly, the AT_1_R displays a somewhat peculiar behavior in terms of its desensitization. Not only is it subject to phosphorylation by other kinases (such as PKA and protein kinase C, PKC) in addition to GRKs [44], but also its phosphorylation is sometimes not even required for desensitization [45]. Thus, it apparently can desensitize through a plethora of different mechanisms and interactions with various other proteins, and what is more, some of the signaling pathways it elicits display different desensitization kinetics from others, e.g., Ca^2+^ transients induced by AT_1_Rs readily and rapidly desensitize, whereas ERK activation and Janus kinase/Signal transducer and activator of transcription (JAK/STAT) signaling emanating from this receptor persist for longer periods of time [46]. Much more has come to light over the past several years about the physiological roles of cardiac βarrs when they mediate G protein-independent signal transduction by the AT_1_Rs in the heart. The first such landmark study was conducted in 2005 and showed, remarkably, that an artificially constructed AT_1A_R mutant (AT1-i2m), incapable of activating G proteins but able to interact with βarrs, led to significantly less myocardial apoptosis and fibrosis, and enhanced cardiomyocyte hypertrophy, bradycardia, and fetal cardiac gene expression upon its exogenous overexpression in cardiomyocytes of transgenic mice *in vivo*, compared to wild type cardiac AT_1_R expressed at similar receptor levels (*B*_max_ values) [47]. In primary cardiomyocytes, the AT_1_R βarr-”biased” agonist AngII peptide analog SII [48] stimulates cardiomyocyte proliferation independently of G proteins [49], but not hypertrophy, which requires G_q/11_ protein signaling [50,51] (Figure 1). In addition, this βarr agonist peptide produces positive inotropic and lusitropic effects in isolated murine cardiomyocytes through GRK6-mediated phosphorylation of the cardiomyocyte AT_1_R and subsequent βarr2 activation [49] (Figure 1). Interestingly, GRK2-mediated phosphorylation of the AT_1_R in cardiac myocytes leads to activation of the other βarr isoform (βarr1), and cardiac βarr1 seems to oppose these positive effects of βarr2 on AT_1_R-elicited contractility and relaxation, *i.e.*, βarr1-mediated signaling results in negative inotropy and lusitropy upon AT_1_R activation in cardiac myocytes [49] (Figure 1). These findings are entirely consistent with specialized roles of the various GRK isoforms described in transfected systems [52], and also with the concept of GRK-induced receptor “barcoding”, *i.e.*, the phenomenon in which different GRK isoforms acting on the very same GPCR induce subsequent recruitment of different βarr isoforms resulting in different downstream signaling events and cellular responses, presumably by phosphorylating the same receptor at different sites/residues [53]. In contrast with isolated murine cardiac myocytes however, SII-activated AT_1_R (*i.e*., AT_1_R-bound βarrs) does not seem to produce any inotropic or chronotropic effects in isolated Langendorff-perfused cardiac preparations, despite the fact that ERK1/2, which presumably mediate the positive inotropic effects of βarr2 in isolated cardiac myocytes, are also activated by AT_1_R-bound βarrs in Langendorff preparations [54]. Thus, it seems that these positive inotropic effects of cardiac βarr2 are strongly cell type- and experimental condition-dependent. Nevertheless, a consensus has emerged, according to which cardiomyocyte-residing AT_1_Rs promote hypertrophy and cardiomyocyte proliferation via βarrs, as well as contractility via (at least) βarr2, whereas cardiac fibroblast-residing AT_1_Rs promote fibrosis and cardiac adverse remodeling via the classical G_q/11_ protein-PKC-Ca^2+^ signaling pathway (Figure 1). Since βarr2 also terminates the G protein-mediated signaling of the AT_1_R, stimulation of cardiac βarr2 activity and/or blockade of cardiac βarr1 activity at the AT_1_Rs of the heart might be sought after for the treatment of post-MI HF and the cardiac hypertrophy and adverse remodeling that accompany this devastating disease. Indeed, a compound analogous to SII, *i.e.*, a βarr-”biased” AT_1_R peptide agonist that selectively activates βarrs while blocking G-protein signaling, TRV120027, has shown very promising results in canine models of acute HF, blocking the undesirable G protein-mediated AT_1_R-induced vasoconstriction, thereby preserving renal function, while, at the same time, enhancing the desirable (in acute HF) βarr-dependent contractility of cardiac myocytes [55], and it is currently under development for the treatment of HF.

Another interesting example of cardiac AT_1_R βarr-mediated signaling is that of the mechanical stretch-activated AT_1_R. A recent study showed that simple mechanical stretch (in the absence of any ligand) can actually activate the AT_1A_R leading to selective βarr recruitment and signaling without concomitant G protein activation [56]. What is more, the authors went on to show in an *ex vivo* murine heart model that this stretch-activated AT_1A_R-elicited βarr signaling resulted in enhanced ERK1/2 and Akt kinase (protein kinase B, PKB) activation, as well as EGFR transactivation, effects believed to mediate enhanced cardiomyocyte survival and protection (inhibition of apoptosis) [56]. In mouse hearts lacking βarrs or AT_1A_Rs, mechanical stretch failed, of course, to produce these responses and led, instead, to enhanced myocyte apoptosis [56]. Thus, it appears that the heart is also capable of responding to acute increases in mechanical stress by activating cardiac βarr-mediated cell survival signals, which again argues in favor of a beneficial and therapeutically desirable physiological role for cardiac AT_1_R βarr-dependent signaling (at least for the cardiac βarr2 isoform-dependent one).

## 5. Other Cardiac GPCRs and βarrs

Unfortunately, and although there is a plethora of other GPCRs expressed in cardiac myocyte membranes, whose signaling also plays important roles in regulation of cardiac function, e.g., glucagon receptors that stimulate contractility, vasopressin receptors (V_1_Rs) and α_1_ARs that promote hypertrophy and cell proliferation, adenosine receptors that regulate heart rhythm, *etc.* [4], the involvement of cardiac βarrs (if any) in their signaling and function specifically in the heart has not so far been investigated. As their importance for cardiac physiology and for heart disease treatments continues to unravel though, investigations of the effects of cardiac βarrs on signaling and function of these GPCRs, as well, are bound to come to light.

## 6. Unanswered Questions on Cardiac βarrs

Clearly, several interesting and important questions about the roles of the two βarrs in cardiac physiology and disease have arisen from the preceding sections, which currently await elucidation in future studies. With regards to β_1_AR signaling and function in the heart, the emerging consensus is that βarr1 is generally detrimental, and βarr2 might actually be beneficial for β_1_AR-regulated cardiac function. βarr1 appears to be cardiotoxic, since (a) it is this cardiac βarr isoform that is responsible for β_1_AR desensitization (G protein uncoupling) and downregulation, processes significantly contributing to the decline of cardiac β-adrenergic and inotropic reserves that underlies the pathophysiology of HF [27]; and (b) the G protein-independent signaling from cardiac β_1_AR it mediates is largely cardiotoxic (inhibition of EGFR transactivation and CaMKII induction leading to cardiac apoptosis, SERCA2a activity lowering leading to reduced contractility, *etc.*) [27] (Figure 1). Coupled with its negative actions in the adrenal glands (stimulation of catecholamine secretion and of aldosterone production and secretion) during HF [27,57–59], which substantially increase the neurohormonal burden of the failing heart, βarr1 seems to be a valid and potentially important therapeutic target in post-MI HF (and maybe also in other heart diseases). Conversely, βarr2 appears to be cardioprotective, since (a) it does not seem to participate in β_1_AR desensitization/downregulation [27]; and (b) the G protein-independent signaling from cardiac β_1_AR it mediates is largely beneficial (EGFR transactivation and ERK activation leading to survival/proliferation, SERCA2a activity enhancement, via SUMOylation, leading to increased contractility, *etc.*) [27] (Figure 1). Also, it does not seem to have significant neurohormonal effects in the adrenals [56–58]. However, whether it is also involved in cardiac β_1_AR-induced CaMKII activation, as βarr1 is [31], is an open question that needs to be addressed in future investigations.

With regards to βarr involvement in β_2_AR signaling and function in the heart, even less is known at present. Do both βarrs desensitize this receptor in the failing heart or one of the two selectively? Do both βarrs recruit PDE4D to the cardiac β_2_AR thereby diminishing its pro-contractile signaling or one of the two selectively (Figure 1) [37,39]? Do both βarrs participate in its purportedly beneficial, anti-apoptotic signaling (e.g., towards ERK activation and NF-κB inhibition) [60,61] in the heart or, again, one of the two selectively? Finally, do the β-blockers that stimulate βarr signaling from the β_2_AR (such as carvedilol) and exert beneficial effects on the failing heart induce binding of both βarrs equally to the cardiac β_2_AR *in vivo* or preferentially induce binding of one βarr over the other [42,62]? Since cardiac βarr1-dependent signaling appears to be damaging and cardiac βarr2-dependent signaling beneficial, preferential induction of βarr2 over βarr1 at the cardiomyocyte β_2_AR by “βarr-biased” β-blockers, like carvedilol, might be one mechanism by which these drugs exert their beneficial effects in the heart. Nevertheless, all these are very important questions that absolutely need to be elucidated in order to fully assess the impact of βarrs on cardiac physiology and their potential as therapeutic targets for treatment of HF and of other heart diseases.

As far as cardiac AT_1_R signaling and function are concerned, important, currently unanswered questions on the roles of βarrs include: (a) whether βarrs are actually involved in regulation of AT_1_R-dependent contractility (and if cardiac AT_1_R is capable at all of signaling to increased contractility to begin with) and whether they oppose each other in that regard, *i.e.*, GRK2/βarr1 block and GRK6/βarr2 promote AT_1_R-dependent contractility (Figure 1), as suggested by *in vitro* studies in cultured cardiomyocytes [49]; (b) whether βarrs differ in their signaling to ERK and Akt activation and EGFR transactivation from the cardiac AT_1_R; (c) whether both contribute to the anti-apoptotic, pro-survival and pro-proliferative (e.g., ERK1/2) signaling of AT_1_R in the heart or one cardiac βarr mediates that signaling more than the other [63]; (d) whether they differ in desensitization (uncoupling) of the classical G_q/11_ protein signaling of the AT_1_R, which is known to result in maladaptive hypertrophy and adverse remodeling in the heart (Figure 1) [50,51], and, finally; (e) whether mechanical stretch preferentially activates one cardiac βarr over the other at the cardiac AT_1_R or both equally [56]. Given that βarr “biased” agonists for the AT_1_R are currently in development for HF [64,65], answering these questions would tremendously aid work towards the full realization of the potential and the prospects of these kind of compounds for cardiovascular therapy.

In summary, the majority of the current literature on βarrs and cardiac βAR and AT_1_R signaling and function seems to overwhelmingly support the notion that βarr2 has (mainly) positive roles in cardiac physiology and function, whereas βarr1 has an overall negative impact on cardiac homeostasis and function, while also opposing several of the beneficial effects of cardiac βarr2. Thus, selective βarr2 stimulation in the heart and/or selective βarr1 inhibition (which leads to βarr2 stimulation indirectly, since the two βarrs always compete with each other inside the cell for binding to any given agonist-activated receptor) might be a valid therapeutic strategy in HF.

## 7. Conclusions and Future Perspectives

The two βarrs were discovered over two decades ago as plain negative regulators of G protein signaling by GPCRs. The realization that they can also act as signal transducers for these receptors, which occurred around the last turn of the century, revolutionized the fields of βarr biology, physiology and pharmacology. Nowadays, they are known to play important roles in function and pathophysiology of almost every organ/system in mammals, including the heart, via their actions on signaling of cardiac GPCRs such as the β_1_AR, the β_2_AR, and the AT_1_R. The accelerating pace at which their roles in the heart are being uncovered has already brought cardiac βarrs to the attention of pharmaceutical scientists and medicinal chemists, and may soon bring them also into the clinic as valid targets for cardiovascular therapy. Before they can be considered for therapeutic targeting in the heart however, full delineation of their effects on cardiac adrenergic, angiotensin and other GPCRs is warranted. With regards to cardiac βARs, a consensus had already begun to emerge: βarr2 appears cardioprotective and βarr1 cardiotoxic. Thus, βarr2 stimulation and/or βarr1 inhibition at cardiac βARs, either directly with pharmacological tools (e.g., small molecules or peptides) or indirectly with biased receptor ligands that bind the cardiac βAR extracellular side and selectively activate βarr2 on its intracellular side, seems to be a good strategy for future cardiovascular drug development. However, there is still a long list of question marks regarding cardiac βarrs and the AT_1_R, and even more questions regarding cardiac βarrs and other GPCRs, not to mention that the puzzle of cardiac βarrs and βARs is still far from being complete, as well. What we already know with certainty is that the two βarr isoforms, once thought of as complementary to each other, are anything but equal or interchangeable when it comes to their actions in the heart. The task of the future investigations in the field of cardiovascular βarrs is thus to fully delineate their differences in heart physiology and disease.
